# Reconstruction and Paleogenetic Characterization of the Genome of Princess Maria Vsevolozha, Wife of Grand Prince Vsevolod the Big Nest

**DOI:** 10.32607/actanaturae.27916

**Published:** 2026

**Authors:** K. V. Zhur, M. V. Leonova, F. S. Sharko, E. D. Pankratova, A. V. Sirenov, D. S. Korobov, M. V. Dobrovolskaya, N. A. Makarov, E. B. Prokhortchouk

**Affiliations:** Federal Research Center “Fundamentals of Biotechnology”, Russian Academy of Sciences, Moscow, 119071 Russia; National Research Center “Kurchatov Institute”, Moscow, 123182 Russia; Saint Petersburg Institute of History, Russian Academy of Sciences, St. Petersburg, 197110 Russia; Institute of Archeology, Russian Academy of Sciences, Moscow, 117292 Russia

**Keywords:** Rurikids, Princess Maria Shvarnovna, Alan origin, ancient DNA, whole-genome sequencing, IBD analysis

## Abstract

Grand Prince of Vladimir Vsevolod the Big Nest and his wife Maria Vsevolozha (Shvarnovna) had twelve children, eight of whom were sons; Grand Prince Alexander Yaroslavich Nevsky was their grandson. Two competing hypotheses exist regarding the origin of Maria Vsevolozha, attributing it to either Ossetian or Czech nobility. Maria Shvarnovna was buried in the Knyaginin Monastery in Vladimir, Russia. Although her remains were reinterred several times, they were never moved from the original burial location. During the 2015 restoration works, skeletal remains, presumably belonging to Maria Shvarnovna, were sampled for paleogenetic analysis in order to determine their origin. More than 144,000 single nucleotide polymorphisms (SNPs) were identified. Their analysis demonstrated that the remains belong to a female carrying mitochondrial haplogroup U4b1a4 and unambiguously confirmed a third-degree biological relationship (great-grandmother–great-grandson) with Prince Dmitry Alexandrovich, the son of Grand Prince Alexander Yaroslavich Nevsky. This result fully corresponds to the genealogical relationships described in medieval chronicles. A multivariate statistical analysis of the genome revealed the two most probable proximal genetic sources of her origin: an Alan component, understood here in a broad cultural-historical sense encompassing populations of the Early and High Middle Ages, and an East Eurasian component. Among medieval populations, the genome of Maria Shvarnovna shows the closest genetic affinity to individuals associated with the SaltovoMayaki archaeological culture, characteristic of the Don forest-steppe region and resulting from the migration of Alan tribes, as well as to individuals from the Kara-Zhygach necropolis (14^th^ century), whose genomes also consist of a dominant Alan and a minor East Eurasian component. Not only do the findings support the Alan hypothesis and reject the Czech origin of Maria Shvarnovna, but they also allow one to confidently assert the reliable genetic identification of early representatives of the Rurikid princely dynasty due to the confirmed biological relationship with her great-grandson Dmitry Alexandrovich.

## INTRODUCTION

For studying the genetic profile of medieval members of the Rurikid dynasty, as wide a range as possible of skeletal remains from burials of the members of this princely lineage need to be included. The main difficulties arise from the current condition of medieval necropolises, which only rarely allow reliable personal identification of burials belonging to the Old Russian elite. The earliest reliable genetic data on medieval Rurikids, obtained from bone tissue samples from the burial site of Prince Dmitry Alexandrovich in the Transfiguration Cathedral of Pereslavl-Zalessky, showed promise for identifying securely attributed remains of other members of the princely family in historical necropolises of North-Eastern Rus’ [1].

One such site is the necropolis of the Knyaginin Monastery in Vladimir, known as the burial place of princesses of the Vladimir princely house [2]. According to the chronicle tradition, the founder of the Knyaginin Monastery was Maria Vsevolozha (Maria Shvarnovna), the wife of Vsevolod the Big Nest and the mother of his twelve children. According to the chronicles, in 1200 Vsevolod founded “a stone church in the name of the Dormition of the Holy Mother of God in the convent of the princess” [3]. In 1201, Maria’s sister, the wife of Prince Yaroslav Vladimirovich, was buried in the monastery; in 1205, the daughter of Maria and Vsevolod, Elena, was interred there [3]; and Maria was buried there in 1206 [4].

The ambiguity of historical records and the complex history of multiple reburials associated with reconstructions of the Dormition Cathedral (Supplementary 1) explain the sustained scholarly interest in the skeletal remains that may belong to Princess Maria and her relatives.

In 2015, during reconstruction works beneath an arcosolium in the wall of the northern aisle of the cathedral, the remains of four individuals were discovered: a woman aged 45–50 years (Individual No. 1), a woman aged 25–30 years (Individual No. 2), a child approximately 9 years old (Individual No. 3), and a young individual of undetermined sex represented only by a fragment of the occipital bone (Individual No. 4) [5]. The initial examination of both the context of the finds and the skeletal material was conducted by S.A. Nikitin, an anthropologist and forensic expert, and T.D. Panova, an archaeologist. [5]. Subsequently, detailed craniological, osteological, and paleopathological studies of three individuals were carried out and a facial reconstruction was performed based on the skull of Individual No. 1, belonging to the older woman. The authors compared individual craniological features of skull No. 1 and demonstrated its morphological affinity with female skulls from the medieval Zmeysky burial ground in North Ossetia, representing the Alan tradition of the 10^th^–14^th^ centuries, which may be viewed as grounds for the hypothesis of an Alan origin. A paleopathological analysis of the skeleton of Individual No. 1 revealed multiple age-related degenerative-dystrophic changes in the spine and on articular surfaces, interpreted by the authors as osteochondrosis and arthrosis with signs of inflammation [5], which could have caused chronic pain.

A mitochondrial genome analysis was also performed, revealing identity of the mitochondrial DNA sequence (U4b1a4) in Individuals No. 1 and No. 3. K.A. Averyanov suggested that Individual No. 1 is Maria Shvarnovna; Individual No. 2 is Anna, the second wife of Vsevolod the Big Nest; and Individual No. 3 is Eudokia, the daughter of Alexander Nevsky [6]. V.I. Merkulov, in contrast, proposed that Individual No. 1 corresponded to the first wife of Alexander Nevsky (daughter of Prince Bryachislav of Polotsk); Individual No. 2, to Vasilisa, the wife of Andrey Alexandrovich of Gorodets (son of Alexander Nevsky); and Individual No. 3, to Eudokia, the daughter of Alexander Nevsky [7, 8].

Confirmation or refutation of the hypothesis attributing Individual No. 1 to Princess Maria has only now become possible following the publication of the whole-genome data from Prince Dmitry Alexandrovich, a confirmed member of the Rurikid dynasty [1].

## EXPERIMENTAL

Ancient DNA (aDNA) was handled in a dedicated clean-room facility at the Federal Research Center of Biotechnology of the Russian Academy of Sciences (K.G. Skryabin Institute of Bioengineering). DNA was extracted from a tooth fragment (incisor) by magnetic separation [9]. Sequencing libraries were prepared using the xGen™ ssDNA & Low-Input DNA Library Preparation Kit (IDT, USA). Target enrichment of the genomic regions of interest was performed using the MyBaits Expert Human Affinities Prime Plus Kit (Daicel Arbor Biosciences). Sequencing was conducted on an Illumina HiSeq 1500 platform (Illumina, USA) in the paired-end mode (2 × 150 bp). Filtering of contaminating DNA reads was performed using BBDuk [10]. Downstream data processing was conducted using the PALEOMIX pipeline (version 1.2.14) [11], including adapter trimming [12] and alignment of reads to the human reference genome (hg19/GRCh37) [13]. Quality filtering, indexing, sorting, and duplicate removal (rmdup) were performed using samtools (version 1.9) [14]. Genotypes were called using PileupCaller with the “–randomHaploid” option. Patterns of post-mortem DNA damage were assessed using MapDamage2 [15]. To determine clustering of the studied sample among published ancient genomes from the Allen Ancient DNA Resource (AADR) panel [16], ADMIXTURE v1.3.0 was used [17]. SNPs were pruned for linkage disequilibrium using PLINK v1.9 (–indep-pairwise 50 5 0.2) [18]. The number of clusters (K) ranged from 4 to 12 (Supplementary 2). Principal component analysis (PCA) was performed using the smartpca tool from the EIGENSOFT package. The list of samples included in the analyses is provided in Supplementary 3. Mitochondrial haplogroups were assigned using HaploGrep [19]. Genome modeling was performed using the qpWave and qpAdm statistical tools with parameter “allsnps: YES”. Genetic relatedness analysis was conducted using IBD-based methods [20] and READ [21]. Phenotypic traits were inferred using the HIrisPlex-S online tool [22, 23]. Sequencing data for the MSh sample (laboratory ID P145) are publicly available at russiangenome.ru/ P145.bam.

## RESULTS AND DISCUSSION

**The results of ancient DNA sequencing of remains presumably belonging to Princess Maria Shvarnovna**

Table S1, Supplementary 4, lists the sequencing data for the DNA library prepared from the genetic material presumably belonging to Princess Maria Shvarnovna. The endogenous DNA content was 1.7% (Fig. S1, Supplementary 4). More than 304 million sequencing reads were generated, yielding 144,938 SNPs. An analysis of mitochondrial markers assigned the sample to haplogroup U4b1a4. Assessment of contamination levels revealed no detectable contamination in the analyzed sample (Tables S2–S3, Supplementary 4). A sufficient number of SNPs enabling probabilistic phenotypic prediction was obtained for the studied DNA sample. With the highest probability, the individual had blue eyes, light hair, and a dark (olive) skin tone (Table S4, Supplementary 4).

**Testing the hypothesis of kinship between Princess Maria Shvarnovna and Prince Dmitry Alexandrovich**

According to genealogical reconstructions of the Rurikid dynasty [24, 25], Maria Shvarnovna is identified as the great-grandmother of Grand Prince Dmitry Alexandrovich (hereafter referred to as DA). Therefore, if the analyzed genome does belong to Princess Maria Shvarnovna, a third-degree biological relationship between this genome and that of Prince DA – previously reconstructed in an earlier study [1] – is expected.

**Fig. 1:**
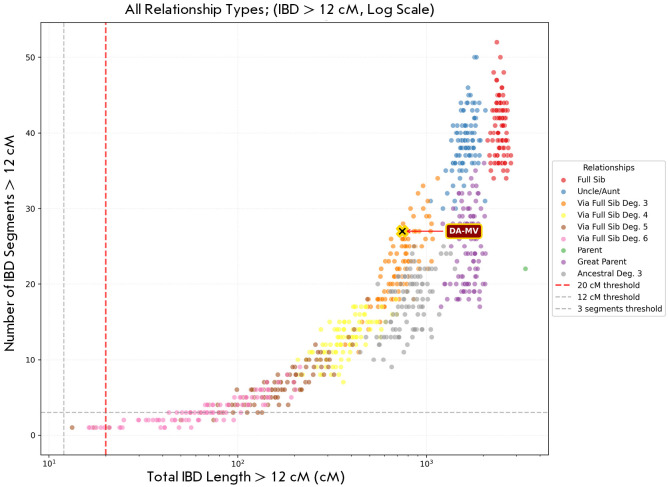
Distribution of the number of IBD segments and the total length of IBD segments longer than 12 cM for pairs of samples with different degrees of relatedness

Verification of the degree of kinship between the two genomes was performed by IBD analysis. Figure 1 shows the distribution of the number of IBD segments and the total length of IBD segments exceeding 12 cM for sample pairs of different degrees of relatedness. A histogram showing the distribution of shared haplotype block lengths is presented in Supplementary 5. The obtained values for the compared genomes correspond to a third-degree relationship, which is consistent with historical records indicating the burial of Prince DA’s great-grandmother in the necropolis of the Knyaginin Monastery. The thirddegree relationship was independently confirmed using the READ tool [21] (Supplementary 6). In the sections below, the analyzed sample is referred to as the genetic material of Princess Maria Shvarnovna (hereafter referred to as MSh).

**Results of PCA**

**Fig. 2:**
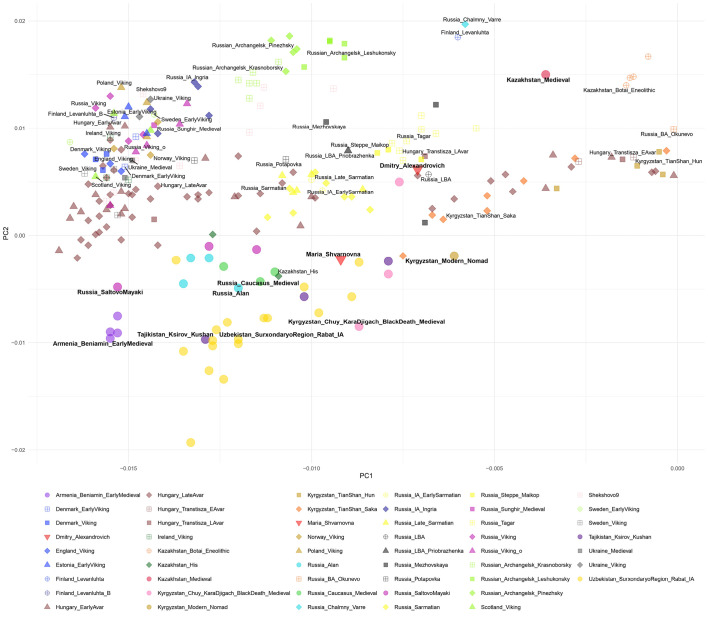
Principal component analysis: projection of the genome of Princess MSh (labeled Maria_Shvarnovna) onto ancient Eurasian populations

**Fig. 3:**
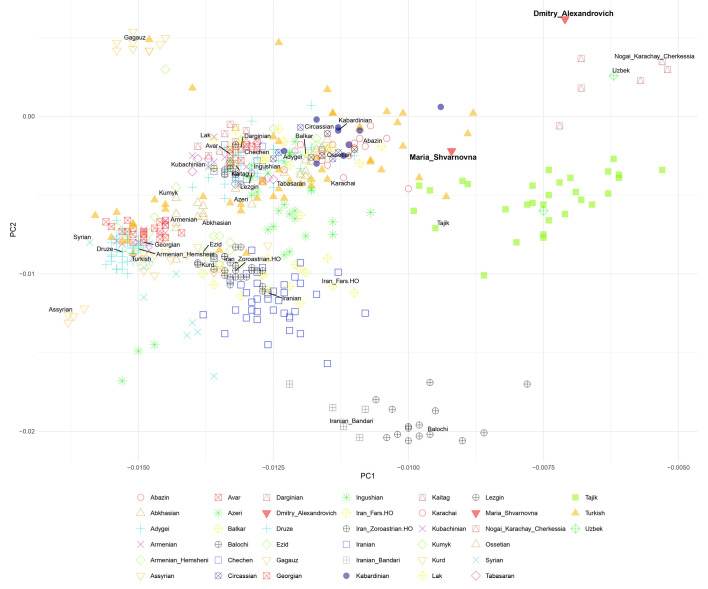
Principal component analysis: projection of the MSh genome (Maria_Shvarnovna) onto modern West Eurasian populations

The genetic affinity of the MSh genome to other ancient (Fig. 2) and modern (Fig. 3) populations was assessed using the principal component analysis (PCA). In the space of ancient genomes, the MSh sample is positioned east of individuals from the medieval Russia_Alan population of North Ossetia dated to 450–850 CE, representatives of the Alan variant of the Saltovo-Mayaki culture (Saltovo_Maiaki) dated to 610–775 cal CE [26], and samples of Russia_Caucasus_ Medieval from the Krasnodar region (Andreyevskaya Shchel), dated to 886–992 cal CE [27].

In close proximity to the MSh genome on the PCA plot are several representatives of the Late Iron Age from southern Uzbekistan (Rabat site in the Surxondaryo region, Uzbekistan_SurxondaryoRegion_Rabat_IA) dated to 150 BCE–50 CE [28], individuals buried in the Christian cemetery of Kara-Zhygach in northern Kyrgyzstan (1338–1339 CE) [29], and an ancient individual from the Ksyrov cemetery in southern Tajikistan dated approximately to the 2^md^ century BCE–1^st^ century CE (Kushan period) [30].

The affinity to the “Kushan” individuals from southern Central Asia is likely explained by their genetic profile, which includes a substantial proportion of a Late Bronze Age steppe component, 15–20% ancestry related to local farmers of southern Central Asia/the Iranian Plateau, and 35–40% ancestry related to Anatolian farmers [30]. In contrast, the genome of Prince DA is significantly shifted eastward relative to that of Princess MSh and clusters in close proximity to Hungarian Avars, an early medieval population of Central Europe.

In the space of modern genomes, the sample of Princess MSh is located outside the Caucasian cluster and occupies an intermediate position between modern Turks and Tajiks (Fig. 3).

These findings can be explained given the fact that modern Turks are highly heterogeneous and represent a complex mixture of ancient Anatolian and Balkan populations, Caucasian/Near Eastern genetic components, and a Central Asian Turkic component in varying proportions depending on the region of residence [31]. Tajiks represent a classic Indo-Iranian admixture, with the majority of their genome derived from Iranian farmer-related ancestry and steppe pastoralists of the Andronovo horizon, with a minor East Asian component [32]. In turn, the genome of Prince DA is positioned closer to the genomes of Nogais from Karachay-Cherkessia, who have previously been shown to differ substantially from most other peoples of the North Caucasus and carry up to 20–50% East Asian ancestry [33].

**Ancestry analysis of Princess MSh using ADMIXTURE**

**Fig. 4:**
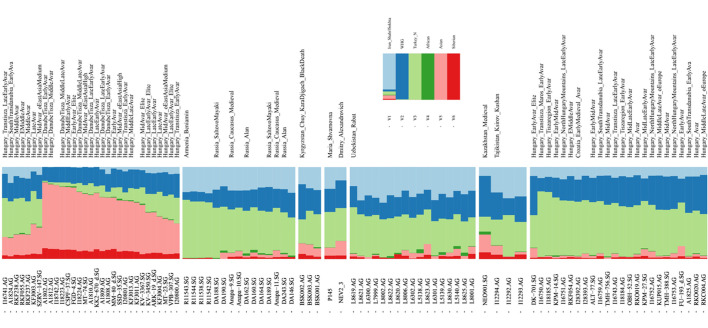
...

An ADMIXTURE analysis was performed to assess the contribution of different “ancestral populations” to the genome of Princess MSh (Fig. 4, Supplementary 2). According to the results for K = 6, the genomes of Princess MSh and Prince DA show similar patterns of ancestry decomposition. However, the Asian component (shown in pink in the plot) is less pronounced in Princess MSh compared to Prince DA, while the blue component is more prominent in Princess MSh. This blue component reaches its maximum representation in the Bronze Age population of the southeastern Iranian Plateau – Iran_ ShahrISokhta.

The Iran_ShahrISokhta population is characterized by a high proportion of ancestry related to the early Neolithic population of Iran (Ganj Dareh) and an absence of ancestry associated with Anatolian Neolithic farmers [34]. The ADMIXTURE plot also includes representatives of ancient populations whose genomes are the closest in ancestral composition to that of Princess MSh. Similar genetic profiles were identified in individuals from the medieval Kara-Zhygach cemetery, representatives of the Late Iron Age of southern Uzbekistan [28], some “Kushan” individuals, and representatives of the Avar Khaganate, present-day Hungary. The latter display pronounced genetic heterogeneity, including individuals with a high proportion of East Eurasian ancestry and either minimal or absent Iranian Neolithic ancestry, as well as individuals with low East Eurasian ancestry and substantial Iranian Neolithic ancestry (Fig. 4).

**Results of outgroup f_3_ statistics**

**Fig. 5:**
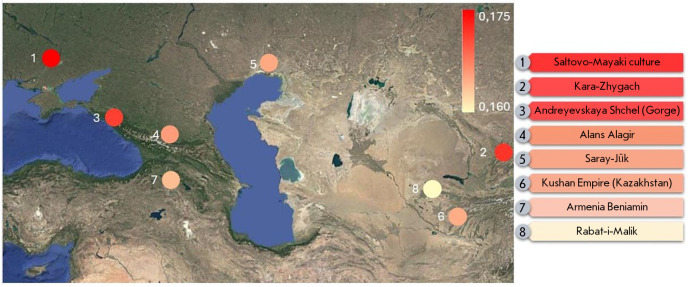
Genetic affinity of the MSh genome to ancient populations assessed using outgroup f3 statistics

Outgroup f_3_ statistics were calculated in the configuration f_3_ (MSh, candidates; Yoruba), where candidates are ancient peoples, whose genomes showed affinity to the genome of Princess MSh based on PCA and the ADMIXTURE analysis. The closest genetic similarity was observed between Princess MSh and representatives of the Alan variant of the Saltovo-Mayaki culture (Russia_SaltovoMayaki) from the Belgorod region of Russia [26], individuals buried in the Christian cemetery of Kara-Zhygach, and samples of Russia_ Caucasus_Medieval (Andreyevskaya Shchel) (Fig. 5, Supplementary 7).

Previous studies have shown that the genomes of Alan representatives of the Saltovo-Mayaki culture are characterized predominantly by a Caucasus/Near Eastern genetic profile, with contributions from steppe ancestry and Ancient North Eurasian (ANE) ancestry, while an East Eurasian admixture is either minimal or absent [26].

Individuals from the Andreyevskaya Shchel cluster, together with representatives of the SaltovoMayaki culture on the PCA plot (Fig. 2) accordingly show similar ADMIXTURE profiles (Fig. 4) dominated by components related to Iranian populations and early European/Anatolian Neolithic farmers, a substantial ANE component, and minor East Asian and Nganasan-related components [27]. Given the dating of these samples, it can be assumed that representatives of both groups may have been part of the Khazar Khaganate.

In turn, individuals from the Kara-Zhygach Christian cemetery display a highly similar genetic profile to those from Andreyevskaya Shchel and the Saltovo-Mayaki culture (Fig. 4), despite their later dating and much more eastern geographic location. These individuals have been shown to be successfully modeled as a mixture of two populations: AR_Xianbei_IA and Alan, with a dominant contribution from the latter [29].

The presence of East Asian and Nganasan-related components in the genomes of these populations does not contradict the anthropological data indicating the presence of Mongoloid traits among populations of the Saltovo-Mayaki culture of the Lower Don and North Caucasus, especially among women [35, 36, 37]. Thus, populations showing the closest genetic similarity to the genome of Princess MSh are characterized by a substantial proportion of Iranian-associated ancestry. Similar proportions of major components are likely to attest to a shared genetic substrate among these groups, representing different regional and chronological manifestations of a single West Eurasian genetic continuum extending from the North Caucasus and the Pontic region to Central Asia and the Tien Shan.

**Modeling the Genetic Origin of Princess MSh**

Modeling of the genetic profile of Princess MSh using qpAdm demonstrated that her genome can be successfully modeled as deriving from a single source represented by individuals from the Kara-Zhygach cemetery, “Kushan” individuals, a medieval sample from Kazakhstan dated to 685–878 cal CE, and representatives of the Alan forest-steppe variant of the Saltovo-Mayaki culture, indicating a high genetic similarity to these populations (Supplementary 8). A model using Russia_Caucasus_Medieval samples did not reach statistical significance. No genetic continuity was detected when modeling with single-source Avar populations (Supplementary 9).

**Fig. 6:**
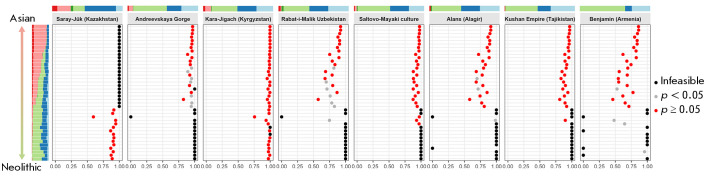
qpAdm modeling of the genome of Princess MSh using two source populations

However, the genomes of various representatives of the Avar Khaganate, present-day Hungary, yielded statistically significant models when used as one of two sources in qpAdm modeling. Robust models were obtained in combination with populations displaying genetic profiles similar to that of Princess MSh: individuals from Kara-Zhygach, “Kushan” individuals, the medieval Kazakhstan sample, and representatives of the Alan forest-steppe variant of the Saltovo-Mayaki culture (Fig. 6, Supplementary 10). Statistically supported models were also obtained using ancient Russia_Caucasus_Medieval samples, medieval Russia_Alan populations from North Ossetia [26], the Armenia_Beniamin_EarlyMedieval population dated to 431–545 cal CE [38], and representatives of the Late Iron Age of southern Uzbekistan.

In addition to Avar populations, statistically supported models were obtained in combinations with ancient populations from Kazakhstan and Kyrgyzstan (Supplementary 11). In total, eight populations yielded valid two-source models for the genome of Princess MSh: representatives of the Alan forest-steppe variant of the Saltovo-Mayaki culture, samples from the Andreyevskaya Shchel burial ground, the medieval Russia_Alan population of North Ossetia, the Armenia_Beniamin_EarlyMedieval population, representatives of the Late Iron Age of southern Uzbekistan, the medieval Kazakhstan_ Medieval (Saray-Jük) sample, individuals from the Kara-Zhygach cemetery, and populations of the Kushan Kingdom. The chronological and historicalethnic context of these populations is provided in Supplementary 12.

Importantly, not all the Avar populations perform equally well in two-source modeling of the genome of Princess MSh, which attests to their substantial genetic heterogeneity [39, 40, 41]. Avar populations with a high proportion of East Eurasian ancestry yielded statistically robust models when combined with “Kushan” individuals, representatives of the SaltovoMayaki culture, ancient samples from Andreyevskaya Shchel, the medieval Russia_Alan population of North Ossetia, the Armenia_Beniamin_EarlyMedieval population, and representatives of the Late Iron Age of southern Uzbekistan. All these populations share a minimal representation of Asian and Siberian components in their genomes. In contrast, the medieval Kazakhstan sample, which carries substantially higher proportions of Asian and Siberian ancestry, generates robust models only in combination with Avar populations in which this component is either absent or minimal. Only for individuals from the KaraZhygach cemetery did 37 out of the 39 Avar populations yield valid models, which is consistent with the results of single-source modeling (Supplementary 8). The smallest number of valid models in combination with Avar populations was observed for the medieval Kazakhstan sample, probably due to its low proportion of Iranian Neolithic ancestry (Fig. 6). Fig. 6. qpAdm modeling of the genome of Princess MSh using two source populations Asian Neolithic Infeasible p < 0.05 p ≥ 0.05 Modeling using ancient Old Russian/Slavic genomes did not produce statistically supported models (Supplementary 11).

**Comparison of the results of genome modeling for Princess MSh and Prince DA**

According to PCA and the ADMIXTURE analysis, the genome of Princess MSh is characterized by a higher proportion of Iranian-related ancestry, whereas the genome of Prince DA exhibits a more pronounced East Eurasian (Asian) component. No statistically supported reasults were obtained when we attempted to model the origin of Princess MSh using three sources that had proved suitable for modeling the genome of Prince DA. In contrast, the genome of Prince DA could be successfully modeled using two sources that proved suitable for modeling the genome of Princess MSh: “Kushan” individuals, the medieval Russia_Alan population, and individuals from the Kara-Zhygach cemetery, in combination with certain Avar populations, although not the same Avar groups that yielded valid models for Princess MSh (Supplementary 13, 14).

Hence, despite the biological relationship between Princess MSh and Prince DA, their genomes differ substantially. This observation further highlights the complex nature of interethnic interactions in the formation of the elite in medieval Rus’ and the diversity of the ethnic origins of women married into the ruling dynasties back then.

## CONCLUSIONS

Our findings confirm the chronicle-based chronology and the great-grandmother–great-grandson relationship between Princess MSh and Prince DA, allowing us to confidently assert the reliability of the genetic identification of ancient members of the Rurikid princely dynasty. The genome of the princess is complex in structure: three major genetic components are associated with Iranian farmers, Anatolian farmers, and Western European hunter-gatherers, while two minor components are represented by East Asian and Siberian hunter-gatherers.

Multivariate statistical characterization of the MSh genome yields the two most probable proximal genetic sources of her origin: an Alan source, understood in a broad cultural-historical sense encompassing populations of the Early and High Middle Ages, and an East Eurasian source. Among medieval populations, the closest genetic relation to the genome of Maria Shvarnovna is observed in representatives of the Alan forest-steppe variant of the Saltovo-Mayaki culture (inhabitants of the Don forest-steppe region), formed as a result of migration of Alan tribes integrated into the Khazar Khaganate, as well as in individuals from the Kara-Zhygach necropolis (14^th^ century), whose genomes also consist of a dominant Alan and a minor East Eurasian component.

These findings support the Alan hypothesis and precludes the Czech origin of Maria Shvarnovna. Notably, phenotypic predictions for Princess MSh (Supplementary 4) are consistent with historical descriptions, including the famous account of the AlanTanaite peoples by Ammianus Marcellinus written at the turn of the 4^th^–5^th^ centuries CE: “Almost all Alans are tall and handsome, their hair is somewhat fair, and their gaze, though not savage, is nevertheless fierce…” [42]. Future studies may produce a more precise localization of the Alan lineage of her ancestry in specific regions of the North Caucasus, the SaltovoMayaki cultural sphere, or other centers of medieval Alan/As populations.

## Abbreviations

aDNA: ancient DNA
SNP: single nucleotide polymorphism
PCA: principal component analysis
