# Erratum to “Reconstruction and Paleogenetic Characterization of the Genome of Princess Maria Vsevolozha, Wife of Grand Prince Vsevolod the Big Nest”

**DOI:** 10.32607/actanaturae.28088

**Published:** 2026

**Authors:** K. V. Zhur, M. V. Leonova, F. S. Sharko, E. D. Pankratova, A. V. Sirenov, D. S. Korobov, M. V. Dobrovolskaya, N. A. Makarov, E. B. Prokhortchouk

## ERRATUM

In the Experimental section, we did not state that the phenotype prediction (pigmentation phenotype) for the ancient individual under study had been performed on a genome with imputed genotypes (imputation carried out using GLIMPSE v1.0.0). At the stage of data preparation for the HIrisPlex-S tool, we made a technical error: genotypes were not converted with respect to strand orientation, which affected the calculation of the phenotypic trait probabilities. The recalculated probabilities of the phenotypic characteristics of the ancient individual are presented in *Table S4*.

**Table S4:** Phenotypic trait prediction results for MSh

Trait	Probability, *P*
Blue eye color	0.026
Intermediate eye color	0.063
Brown eye color	0.912
Light brown hair color	0.042
Brown hair color	0.526
Red hair color	0.000
Black hair color	0.432
Light hair color	0.090
Dark hair color	0.910
Very pale skin	0.014
Pale skin	0.114
Intermediate skin	0.697
Dark skin	0.170
Dark to black skin	0.005

This correction concerns the pigmentation phenotype prediction only and does not affect the data used for identification, kinship analysis, or assessment of the genetic ancestry of the individual. We apologise for this error.

